# Neurofilament light chain as a marker of peripheral nerve damage in vasculitic neuropathy? A cross‐compartmental correlation analysis in patients undergoing nerve biopsy

**DOI:** 10.1111/bpa.70038

**Published:** 2025-09-11

**Authors:** Simon Streit, Jenny Meinhardt, Niclas Gimber, John Kestenbach, Christin Siewert, Jan Schmoranzer, Christian Meisel, Klemens Ruprecht, Frank L. Heppner, Péter Körtvélyessy, Werner Stenzel

**Affiliations:** ^1^ Department of Neuropathology Charité—Universitätsmedizin Berlin, Corporate Member of Freie Universität Berlin and Humboldt‐Universität zu Berlin Berlin Germany; ^2^ Advanced Medical BioImaging Core Facility—AMBIO Charité—Universitätsmedizin Berlin Berlin Germany; ^3^ Labor Berlin—Charité Vivantes GmbH Berlin Germany; ^4^ Department of Neurology Charité—Universitätsmedizin Berlin, Corporate Member of Freie Universität Berlin and Humboldt‐Universität zu Berlin Berlin Germany; ^5^ German Center for Neurodegenerative Diseases (DZNE) Berlin Germany; ^6^ German Center for Neurodegenerative Diseases (DZNE) Magdeburg Germany

**Keywords:** biomarker, histopathology, nerve biopsy, neurofilament light chain (NfL), vasculitic neuropathy, vasculitis

## Abstract

Vasculitic neuropathy remains challenging to diagnose and monitor because of its heterogeneous clinical and laboratory presentation. Blood‐based biomarkers indicating nerve damage could serve as an additional diagnostic tool to ensure early diagnosis, precise therapeutic monitoring, and a more targeted anti‐inflammatory treatment. A potential marker for this purpose is neurofilament light chain (NfL), a marker of neuroaxonal damage that is used as a biomarker in several diseases of the central and peripheral nervous system. NfL has also been suggested to reflect disease activity in patients with vasculitic neuropathy. However, its biodynamics and link to degeneration of peripheral nerve tissue remain unconfirmed. To investigate the usefulness of NfL as a marker of peripheral nerve damage in this context, we retrospectively assembled a cohort of 35 patients undergoing sural nerve biopsies (including patients with vasculitic neuropathy and other neuropathies). We then measured NfL in serum samples cryoarchived at the time of biopsy and correlated NfL levels with histological parameters. For our histological analysis, we quantified parameters of acute axonal degeneration and chronic axonal loss using a combination of manual, threshold‐based, and supervised learning‐based analyses. We found a significant positive correlation between parameters of acute axonal degeneration and serum‐NfL levels that persisted after adjusting for age and concomitant central nervous system disease. We did not find a similar correlation with parameters of chronic axonal loss quantified in nerve biopsies. These findings support the value of NfL as a marker for acute axonal degeneration in patients with vasculitic neuropathy.

## Graphical abstract

To assess neurofilament light chain (NfL) as a biomarker of peripheral nerve damage, we correlated serumNfL concentrations with histopathological parameters from matched sural nerve biopsies. SerumNfL levels correlated with markers of acute axonal degeneration, but not with parameters reflecting chronic axonal loss.

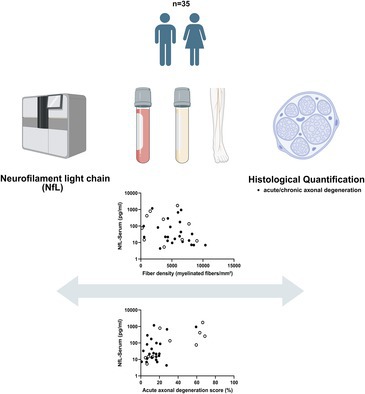

## 1 INTRODUCTION

Neuropathy is a common and painful feature in patients with vasculitis that can present early during the course of the disease and can manifest with or without any other organ involvement [1, 2]. Diagnostic evaluation, disease monitoring as well as therapeutic monitoring are currently challenging [1, 2, 3].

While it is of utmost importance to achieve early diagnosis and prevent persisting and progressive nerve damage, identifying vasculitic neuropathy can be difficult [1, 2]. Nerve biopsy is the gold standard to detect vasculitic neuropathy, but it can cause complications such as wound infection, persistent pain, or permanent loss of sensory function in the corresponding sensory domain [4]. In addition, a firm diagnosis cannot be made in up to 55% of cases because pathognomonic histopathological features such as vessel wall destruction are missing in the biopsy tissue [5, 6].

Once diagnosed, monitoring treatment response is equally challenging (especially in cases of nonsystemic vasculitis), as it is primarily based on clinical exams tracking prolonged nerve recovery [1, 3]. At the same time, the utility of established biomarkers such as C reactive protein (CRP) to monitor disease activity is limited because they indicate systemic inflammation rather than nerve damage, thus capturing only parts of the clinical picture [1, 3].

New biomarkers indicating the extent and nature of nerve damage in patients with vasculitis could help to overcome these barriers, improve diagnostic sensitivity, and aid with a more targeted anti‐inflammatory treatment with fewer side effects [7]. Specifically, they could help clinicians decide when to taper and stop immunosuppressive treatment [7].

Neurofilament proteins are promising candidates for such biomarkers, because they are neuron‐specific and are being released into serum and cerebrospinal fluid (CSF) after neuronal injury [8, 9]. With novel assays detecting neurofilaments in the bloodstream, there has been a particular interest in the neurofilament light chain subunit (NfL) of neurofilaments [10, 11, 12, 13]. NfL assays have been shown to detect hidden epitopes that become available only after neuronal degradation [10]. Consequently, NfL levels have been investigated as a diagnostic marker, prognostic marker, and a marker of disease activity in a variety of diseases of the central and peripheral nervous system, including multiple sclerosis, frontotemporal dementia, amyotrophic lateral sclerosis, spinal muscular atrophy, and Guillain‐Barré syndrome [10, 13, 14, 15, 16, 17, 18, 19, 20, 21].

Specifically regarding patients with vasculitic neuropathy, a recent study by Bischof et al. has shown elevated serum‐NfL levels (sNfL) in acute disease that decreased with remission [7]. However, studies directly linking elevated sNfL levels with peripheral nerve damage are currently scarce [18]. To our knowledge, no study linking histologically proven nerve damage with sNfL levels has been conducted yet. Hence, confounding factors and biodynamics in the context of peripheral nervous system disease remain poorly understood, limiting the use of NfL in clinical practice [13].

To better characterize the relationship between nerve damage and sNfL levels, and investigate its usefulness as a clinical marker in the diagnosis and monitoring of vasculitic neuropathy, we retrospectively identified matched nerve biopsy‐ and serum samples in a cohort of patients that underwent sural nerve biopsy (including patients with vasculitic neuropathy and other neuropathies). We hypothesized that sNfL levels would show a strong positive correlation with axonal degeneration as detected in nerve biopsies.

## 2 METHODS

### 2.1 Ethics and consent

Consent to use clinical data and biomaterials for research purposes is based on the Berlin Hospital Law and written consent at the time of hospital admission. The study was approved by the Charité Ethics Committee (EA/269/21).

### 2.2 Patient recruitment

To identify patients with a diagnosis of vasculitis and matching biosamples, we performed a combination of keyword, diagnosis, and procedure searches in our clinical and neuropathological databases. To cover a wide spectrum of nerve damage severity, we included patients with biopsy results indicating vascular inflammation as well as additional disease entities with matching biosamples available.

We included patient samples taken 30 days prior or post sural nerve biopsy. Clinical data was extracted from clinical reports at the time of biopsy as well as the latest follow‐up report available. Main data points were age, sex, clinical diagnosis, and pathological diagnosis. To present additional patient characteristics that could act as potential confounders for NfL levels, we also collected data on the disease timecourse as well as the interval between biosample collection and nerve biopsy, and screened medical records for concomitant central nervous system (CNS) disease.

The disease timecourse was defined as the interval between disease onset or exacerbation and biosample collection (via lumbar puncture and blood draw). It was categorized as “acute” (biosample collection less than 1 week after onset or exacerbation), “subacute” (1–8 weeks), or “chronic” (>8 weeks). CNS disease was classified based on its relevance for sNfL levels at the time of biopsy.

We collected cryoarchived biosamples from serum, CSF (to control for NfL release from the central nervous system) and nerve (as an additional layer of NfL analysis).

### 2.3 NfL measurements

**FIGURE 1:**
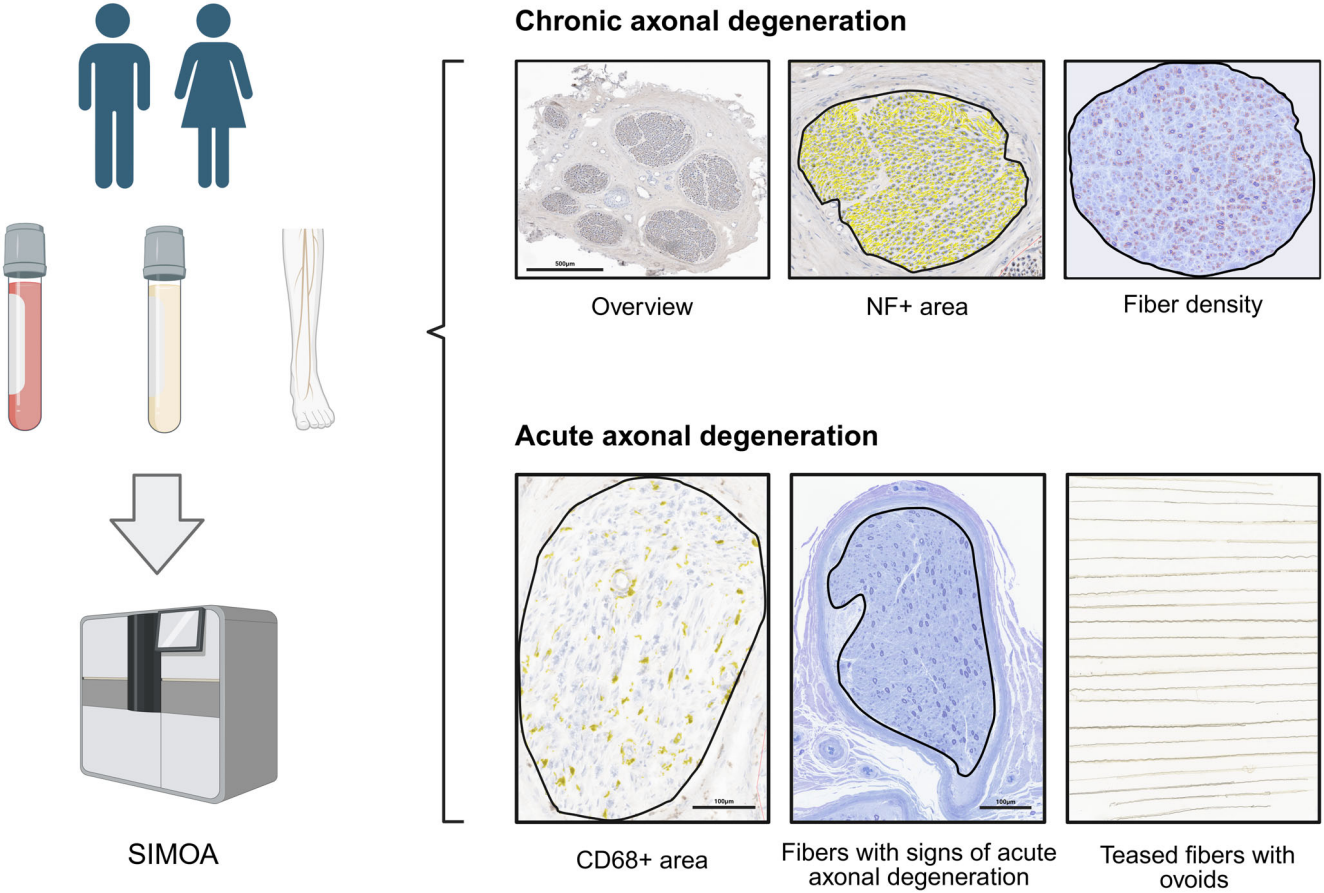
Analysis workflow. NfL levels in serum, cerebrospinal fluid, and nerve tissue from sural nerve biopsies were analyzed using the Single Molecule Analysis (SIMOA) platform. Additionally, nerve tissue was assessed histologically to determine chronic axonal loss as well as acute axonal degeneration. To quantify chronic axonal loss, we digitized slides stained for neurofilaments (NF70; brown) and semithin sections stained with methylene blue. We then performed image analysis on individual nerve fascicles in transversely sectioned nerves. Neurofilament staining (yellow overlay) and myelinated fibers (orange overlay) were detected using automated segmentation approaches. Acute axonal degeneration was assessed using three parameters: (1) macrophage infiltration, quantified as fascicular area covered by CD68+ staining in transverse and longitudinal sections (bottom left); (2) manual counts of fibers showing morphological signs of acute axonal degeneration (bottom center); and (3) the fraction of teased fibers with ovoid formations (bottom right; fiber length: 5 mm).

We measured NfL levels in serum, CSF, and sural nerve biopsy samples using the Single Molecule Analysis (SIMOA) platform according to standard protocol (Figure 1) [22]. For detection of NfL levels in nerve tissue, we collected about 20 cryosections à 10 μm per patient, homogenized the tissue in Tris‐buffered saline, and measured NfL levels in the cell‐free aliquot after centrifugation. To account for mass differences, we also measured the total protein content in these aliquots using a bicinchoninic acid assay (BCA) and expressed NfL levels as % of the total protein fraction.

To account for potential confounding factors, we calculated age‐specific *Z*‐scores for sNfL levels [23] and calculated sNfL to CSF‐NfL ratios to account for any effect of degradation of central nervous system tissue on sNfL levels [17].

Four samples in the tissue dataset (P2, P3, P14, and P21) and four samples in the CSF dataset (P7, P14, P21, and P29) exhibited fluorescence values exceeding the upper limits of detection. As a result, these values were capped at their respective maximum validated detection limits: 7800 pg/mL for tissue and 25,000 pg/mL for CSF. A sensitivity analysis was conducted to evaluate the impact of this capping on our results. The analysis demonstrated that, while higher assumed CSF values led to reduced statistical significance, the overall findings remained robust—except under very high assumptions (see Supplementary Material 5).

### 2.4 Histological workup

In general, we utilized slides prepared during standard diagnostic workup, digitized slides using the Aperio GT 450 DX image scanner (Leica Biosystems, Wetzlar, Germany) and performed quantitative analysis in Qupath, Image J, Aperio Image Scope, or Python. Each automated image analysis was validated in a blinded semiquantitative assessment (Supplementary Material 1). Each manual analysis was performed blinded to any NfL measurements.

Specifically, we utilized cryosections stained for NF70 and CD68, 2.5% glutaraldehyde (GA)‐fixed semithin sections stained with methylene blue and teased fibers stained with *p*‐phenylenediamine (PPD). All procedures were performed according to standard local laboratory protocols.

### 2.5 Chronic axonal loss

#### 2.5.1 Area covered by neurofilament staining

Chronic axonal loss was assessed in sections stained for neurofilament (NF70; Dako; Glostrup, Denmark; M0762). Regions of interest (ROIs) were drawn manually, marking all nerve fascicles in transverse sections. Consecutive sections were studied to ensure sufficient quality of each fascicle for analysis. In the next step, the area covered by NF70 staining was quantified using a custom‐written ImageJ macro [24, 25]. Color deconvolution was applied to isolate NF‐positive staining. Small structures were enhanced using a Laplacian of Gaussian filter, followed by Otsu binarization. Detection was restricted to objects between 20 and 30,000 μm^2^. The total area of NF70‐positive staining within the marked fascicular regions of a transverse nerve section was calculated and expressed as a percentage of the total fascicular area.

#### 2.5.2 Density of myelinated fibers

As an additional indicator of chronic axonal loss, we quantified the density of myelinated fibers in 1 μm‐thick transverse semithin sections stained with methylene blue. We measured fiber density by applying a supervised learning‐based morphometry approach published by Ono et al. [26]. We manually reviewed fascicle and fiber detection and excluded fascicles with inaccurate automated detection. The density of myelinated fibers was calculated as the number of myelinated fibers per fascicular area (in mm^2^).

### 2.6 Acute axonal degeneration

#### 2.6.1 Fibers with morphological signs of acute axonal degeneration

Two experienced neuropathologists (SST + JM) manually counted fibers with morphological signs of acute axonal degeneration such as myelin collapse with grayish violaceus discoloration of myelinated axons and disappearance of proper axonal tissue in transverse semithin sections (Supplementary Material 4). To minimize type I error, only fibers with unambiguous features of acute axonal degeneration were included. After a first round of counting, fascicles with discrepant results were reevaluated in a joint session. If consensus could not be reached, a third neuropathologist (WS) was consulted to make a final judgment within the range of the initial counts.

#### 2.6.2 Teased fibers with ovoids

In a subset of patients, teased fiber preparations or additional tissue fixed in 2.5% GA were available (*n* = 14). For quantification, we counted the number of fibers with ovoid formations (as a measure of Wallerian degeneration). The number of teased fibers with ovoids was put in relation to the total number of assessable fibers (20–53 fibers per case).

#### 2.6.3 Macrophages

For quantification of macrophage infiltration, we quantified CD68+ staining (Dako; Glostrup, Denmark; M0814) in both transverse and longitudinal sections using polygonal ROIs and a tailored image analysis using rolling ball reduction, automated thresholding (“Yen”) and despeckle filtering [27]. Transverse and longitudinal datasets were analyzed separately to account for potential differences arising from sectioning orientation. For each dataset, the CD68+ area was calculated as a fraction of the total fascicular area per nerve section and expressed as a percentage of the dataset's maximum value. Finally, the normalized percentages from transverse and longitudinal analyses were averaged to yield a single value representing total macrophage infiltration.

### 2.7 Data analysis

To ensure robust quantification of our histological endpoints—acute axonal degeneration and chronic axonal loss—we based our analyses on multiple complementary parameters per endpoint.

For acute axonal degeneration, three parameters were quantified: (1) the number of degenerating axons per fascicular area, (2) the fraction of teased fibers with ovoids, and (3) the percentage of fascicular area covered by CD68+ staining. To avoid overemphasizing any single parameter, offset potential technical limitations inherent to each analysis, and improve statistical efficiency (particularly for parameters with limited sample availability, such as teased fiber preparations), these metrics were integrated into a composite score.

To achieve compatible metrics for composition, both the number of degenerating axons per fascicular area and the number of teased fibers with ovoids were normalized to the maximum value within their respective datasets and expressed as a percentage of maximum (% max). The CD68+ staining values were already normalized to % max, as described previously. The composite score was then calculated by averaging these three normalized values, yielding a single % max score representing the degree of acute axonal degeneration per sample. Cross‐correlation analysis revealed positive correlations between the individual parameters (Supplementary Material 1E). Separate analyses and visualizations for each parameter prior to building composites are provided in Supplementary Materials 2 and 3.

For chronic axonal loss, we assessed two metrics: (1) the fascicular area covered by NF70 staining (capturing both myelinated and unmyelinated fibers) and (2) the density of myelinated fibers per fascicular area quantified from semithin sections. Since these two parameters did not correlate (Supplementary Material 1C) and may reflect distinct aspects of chronic axonal loss, we chose to report them separately.

### 2.8 Statistics

Statistical analysis was conducted in Graphpad Prism (Version 9.5.1; Boston, USA). Sample size was determined by the number of biosamples available. To account for non‐normally distributed sNfL data, we performed Spearman rank correlation analysis. We defined a correlation coefficient of 0.5 as the cutoff for a moderately strong and clinically meaningful correlation [28]. We used one‐tailed *t*‐tests for validation of our image analyses, assuming a positive correlation between semiquantitative and quantitative assessments. For hypothesis testing, we performed two‐tailed analyses.

## 3 RESULTS

### 3.1 Patient characteristics

We assembled a cohort of 35 patients (23 men, 12 women) who had undergone sural nerve biopsy and from which at least one biosample (serum, CSF, or nerve tissue) from the time of biopsy was available for further analysis. Their clinical characteristics are presented in Table 1.

**TABLE 1:** Patient characteristics.

Study‐ID	Age	Sex	Clinical diagnosis	Neuropathological diagnosis	Timecourse	∆*d* to biopsy	CNS disease
P1	54	f	Immuncomplex vasculitis	Perivasculitis	Subacute	−1	No
P2	75	m	Hepatitis B‐associated vasculitis	Vasculitis	Subacute	6	No
P3	48	f	Systemic sclerosis and vasculitic neuropathy	Perivasculitis	Acute	16	No
P4	73	m	Vasculitic neuropathy	Vasculitis	Subacute	0	No
P5	53	m	ANCA‐associated vasculitis with polyneuropathy	Vasculitis	Chronic	5	No
P6	58	f	Vasculitic polyneuropathy	Vasculitis	Chronic	−7	No
P7	32	f	Acute motoric axonal neuropathy	Perivasculitis	Subacute	8	Yes
P8	62	f	Suspected vasculitic neuropathy	Mild axonal neuropathy	Chronic	−2	No
P9	66	m	Suspected vasculitic neuropathy	Perivasculitis	Subacute	3	Yes
P10	55	f	Suspected isolated vasculitic neuropathy	Perivasculitis	Chronic	1	No
P11	82	f	Unclear inflammatory polyneuropathy	Perivasculitis	Subacute	4	No
P12	61	m	Unclear axonal polyneuropathy	Perivasculitis	Chronic	−2	Unclear
P13	65	m	Demyelinating polyneuropathy	Neuritis	Subacute	2	No
P14	26	f	Suspected autoimmune encephalitis and polyneuropathy	Neuritis	Subacute	8	Yes
P15	65	m	Axonal neuropathy, most likely because of diabetes	Neuritis	Subacute	16	Unclear
P16	41	m	Small fiber neuropathy	No pathological findings	Chronic	16	No
P17	76	f	Amyotrophic lateralsclerosis	No pathological findings	Chronic	2	Yes
P18	35	m	Polyneuropathy	Mild axonal neuropathy	Acute	6	Unclear
P19	71	m	Chronic inflammatory demyelinating polyneuropathy (CIDP)	Mild axonal neuropathy	Subacute	4	No
P20	75	f	Mixed polyneuropathy	Mild axonal neuropathy	Subacute	9	No
P21	37	m	Unclear myelitis and encephalitis	Mild axonal neuropathy	Subacute	6	Yes
P22	50	f	Muscular dystrophy	Mild axonal neuropathy	Chronic	6	No
P23	60	m	Unclear polyneuropathy	Mild axonal neuropathy	Chronic	0	Yes
P24	48	m	Diabetes mellitus polyneuropathy	Mild axonal neuropathy	Chronic	7	No
P25	44	m	Unclear axonal polyneuropathy	Mild axonal neuropathy	Chronic	1	No
P26	26	m	Cerebellar ataxia	Mild axonal neuropathy	Chronic	3	Unclear
P27	57	f	MGUS‐associated CIDP	Mild axonal neuropathy	Chronic	1	No
P28	75	m	CIDP versus paraproteinemic polyneuropathy	Severe axonal neuropathy	Subacute	21	No
P29	60	m	Guillian‐Barré syndrome	Severe axonal neuropathy	Chronic	−16	Yes
P30	32	m	Chronic inflammatory demyelinating polyneuropathy (CIDP)	Severe axonal neuropathy	Chronic	25	No
P31	47	m	Suspected toxic and diabetic neuropathy	Severe axonal neuropathy	Chronic	12	No
P32	70	m	Paraneoplastic neuropathy	Severe axonal neuropathy	Chronic	4	No
P33	63	m	Suspected chronic inflammatory axonal polyneuropathy (CIAP)	Severe axonal neuropathy	Chronic	1	Yes
P34	67	m	Unclear polyneuropathy	Severe axonal neuropathy	Chronic	−1	No
P35	80	m	Amyotrophic lateralsclerosis	Severe axonal neuropathy	Chronic	6	Yes

*Note*: Patients 1–6 had a definite diagnosis of vasculitis. Medical records of P7–P10 were highly suggestive of vasculitis. Disease timecourse was defined based on the interval between disease onset or exacerbation and sample collection (cerebrospinal fluid [CSF]/serum). Δ*d* to biopsy illustrates time between collection of biosamples and nerve biopsy (positive values indicating sample collection prior to biopsy). No = no CNS disease in medical records or unlikely impact on serum‐NfL (sNfL) levels at the time of biopsy (e.g., migraine or past history of stroke). Yes = CNS disease suggesting sNfL release (e.g. HIV encephalitis). Unclear = uncertain relevance for sNfL levels at time of biopsy (e.g., subsequent medical records indicated a diagnosis of Parkinson's disease). Among nine patients with relevant CNS disease, five patients had a diagnosis of encephalitis or myelitis (P7, P9, P14, P21, and P23), two patients a diagnosis of amyotrophic lateralsclerosis (P17 + P35), one patient had a diagnosis of Parkinson's disease (P12) and for one patient (P29), CNS disease was inferred based on CSF‐NfL levels, although the medical records did not provide a clear diagnosis of an underlying CNS condition.

Abbreviations: ANCA, antineutrophil cytoplasmatic antibodies; CNS, central nervous system; MGUS, monoclonal gammopathy of undetermined significance. HIV, Human Immunodeficiency Virus.

Patients in our cohort presented with a range of different underlying conditions (Table 1). Medical records indicated a definite or probable diagnosis of vasculitis in 10 patients; the remaining 25 patients had other diagnoses resulting in neuropathy. Out of the 10 patients with vasculitis, 6 patients showed an acute or subacute course of disease at the time of biopsy and 5 fulfilled histological criteria of leucocytoclastic vasculitis. Most non‐vasculitis patients had a histological diagnosis of axonal neuropathy (ranging from minimal to severe) and did not show any signs of inflammation in their biopsy (Table 1).

The median time between collection of serum/CSF samples and biopsy was 4 days. In nine patients, medical records or very high levels of CSF‐NfL indicated relevant CNS disease (Table 1).

### 3.2 Serum‐NfL levels do not correlate with chronic axonal loss but with acute axonal degeneration

**FIGURE 2:**
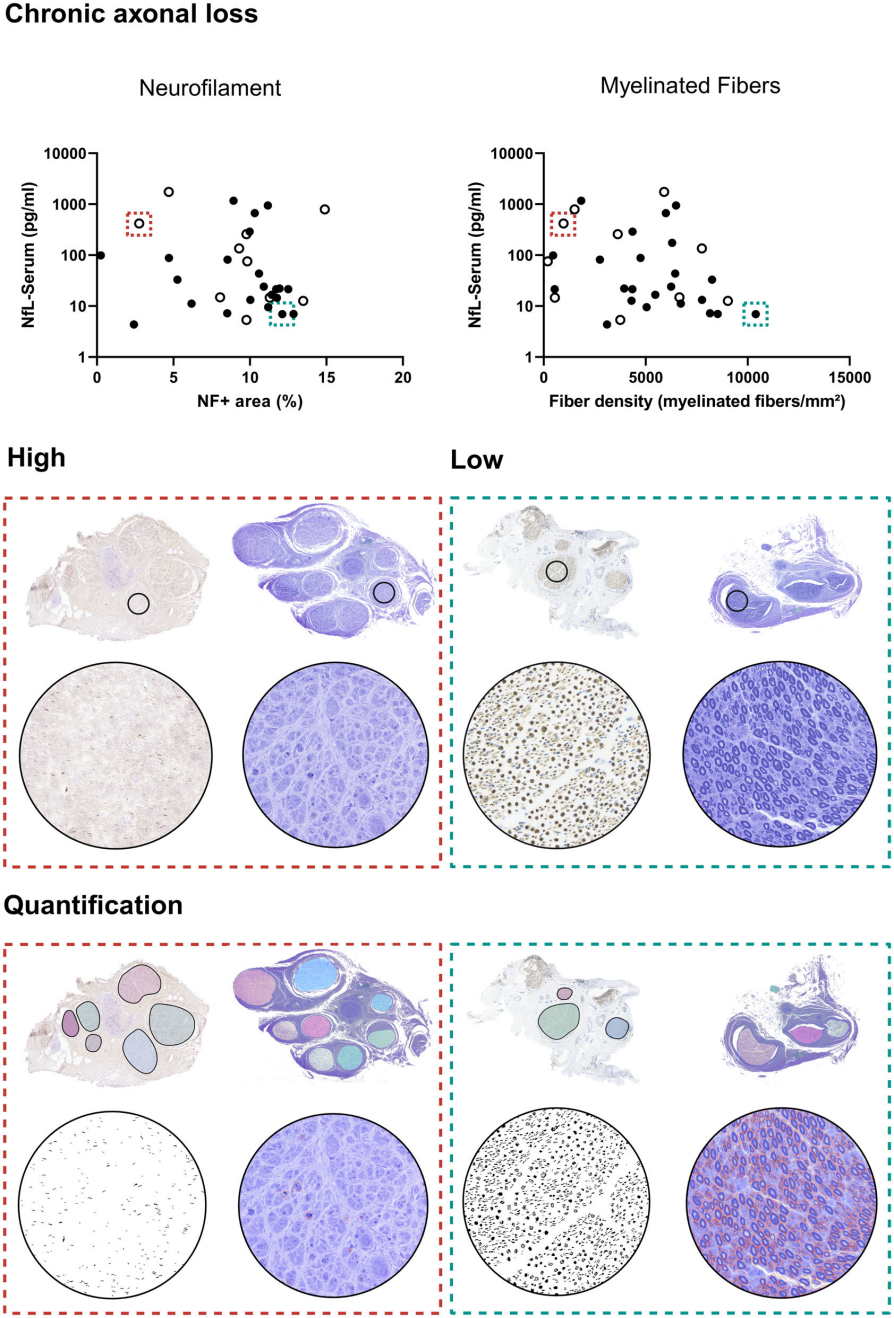
Neurofilament light chain (NfL) levels in serum and nerve tissue do not correlate with parameters of chronic axonal loss. There is no significant correlation between the % of fascicular area covered by neurofilament staining in nerve biopsies and serum‐NfL (sNfL) levels (*r* = −0.2 [−0.5 to 0.1]; *p* = 0.23; *n* = 32). There is also no significant correlation between the density of myelinated fibers in nerve biopsies and sNfL levels (*r* = −0.3 [−0.6 to 0.1]; *p* = 0.08; *n* = 33). Dashed green lines highlight a patient sample with a low degree of chronic axonal loss as detected in sections stained for NF70 (images on the left; brown) and semithin sections stained with methylene blue (images on the right). Dashed red lines highlight a patient sample with a high degree of chronic axonal loss. The bottom images illustrate the detection of nerve fascicles and quantification of NF+ area (left image in each rectangle; detected area displayed in black) as well as the detection of myelinated fibers (images on the right; automatically detected fibers are marked with orange rectangles). Unfilled dots represent data points from patients with a clinical diagnosis of vasculitis.

We first tested whether there was a correlation between parameters of chronic axonal loss and NfL levels. Spearman rank correlation neither indicated a significant correlation between the % fascicular area covered by neurofilament staining and sNfL levels (*r* = −0.2 [−0.5 to 0.1]; *p* = 0.23; *n* = 32) nor between the density of myelinated fibers and sNfL levels (*r* = −0.3 [−0.6 to 0.1]; *p* = 0.08; *n* = 33) (Figure 2). Accordingly, there was no significant correlation between these two parameters and NfL levels in nerve tissue (Neurofilament+ area: *r* = −0.2 [−0.5 to 0.2], *p* = 0.3, *n* = 31; density of myelinated fibers: *r* = −0.3 [−0.6 to 0.04], *p* = 0.07, *n* = 31) (Supplementary Material 3).

**FIGURE 3:**
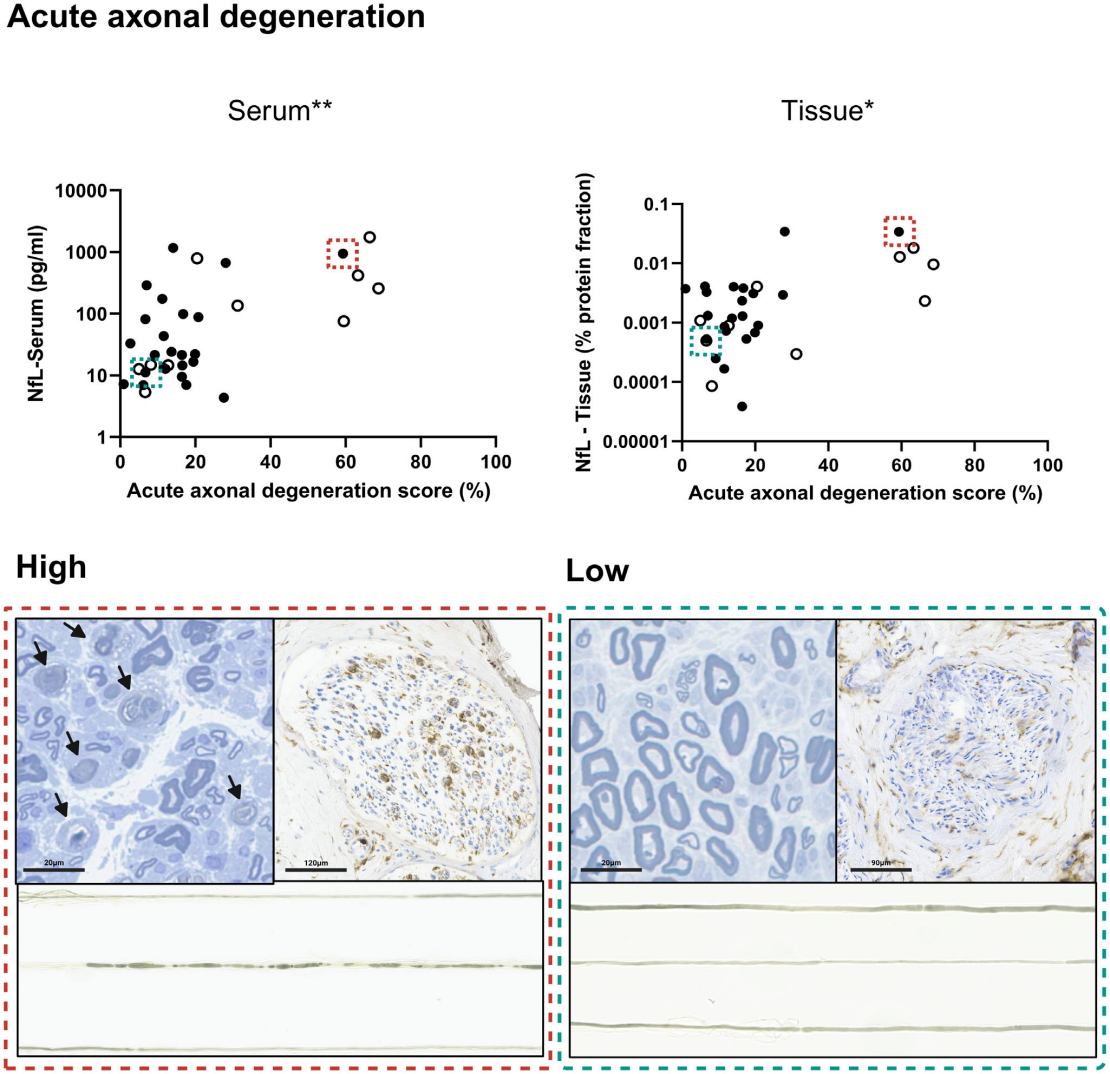
Serum‐ and nerve tissue‐neurofilament light chain (NfL) levels correlate with acute axonal degeneration. There is a significant correlation between acute axonal degeneration scores and NfL levels in serum and nerve tissue (Spearman rank correlation; *n*_serum_ = 34, *n*_Tissue_ = 32; *r*_serum_ = 0.5 [0.2–0.7], *r*_Tissue_ = 0.4 [0.1–0.7]; *p*_serum_ = 0.002, *p*_Tissue_ = 0.02). Dashed lines in green highlight a patient sample with a low degree of acute axonal degeneration. Dashed lines in red highlight a patient sample with a high degree of acute axonal degeneration. Acute axonal degeneration was measured by building a composite score that combines the density of fibers with morphological signs of acute axonal degeneration in semithin sections (images on the upper left; fibers with signs of acute axonal degeneration are marked with arrows), the extent of macrophage infiltration (CD68‐staining; brown; upper right images) and the fraction of fibers with ovoids in teased fiber preparations (lower images; image width = 1 mm; central nerve fiber on the left displays signs of fragmentation/ovoids). Unfilled dots represent data points from patients with a clinical diagnosis of vasculitis. *p < 0.05; **p < 0.01.

Spearman rank correlation, however, did indicate a significant positive correlation between acute axonal degeneration scores from nerve biopsies and NfL levels in serum (*r* = 0.5 [0.2–0.7]; *p* = 0.002; *n* = 34) and nerve tissue (*r* = 0.4 [0.1 to 0.7]; *p* = 0.02 *n* = 32) (Figure 3).

### 3.3 Adjustment for potential confounding factors

**FIGURE 4:**
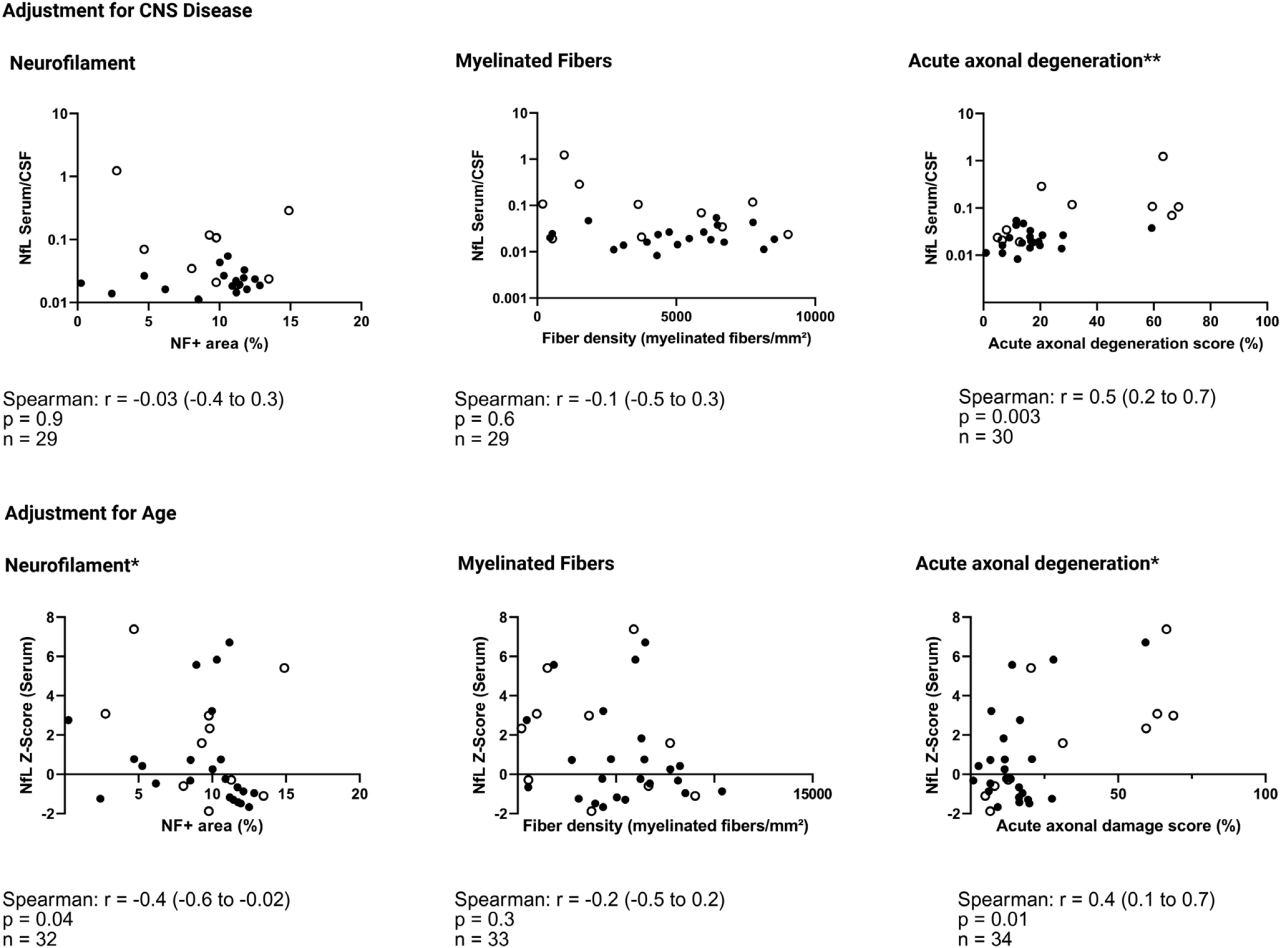
Adjustments for confounding factors. To adjust for central nervous system (CNS) damage, we calculated serum‐neurofilament light chain (sNfL) to cerebrospinal fluid (CSF)‐neurofilament light chain (NfL) ratios. There was no significant correlation between NfL ratios and parameters of chronic axonal loss (indicated by neurofilament positive fascicular area and the density of myelinated fibers). There was, however, a significant correlation between NfL ratios and acute axonal degeneration (indicated by a score combining CD68+ staining, the density of fibers with morphological features of acute axonal degeneration and the fraction of teased fibers with ovoids). To adjust for age, we calculated age‐specific *Z*‐scores for our serum samples. After adjustment for age, the significant correlation between serum‐NfL (sNfL) *Z*‐scores and acute axonal degeneration scores persisted. Parameters of chronic axonal loss showed discrepant results with no significant correlation between sNfL *Z*‐scores and the density of myelinated fibers, but a significant correlation between neurofilament positive fascicular area and sNfL *Z*‐scores. Unfilled dots represent data points from patients with a clinical diagnosis of vasculitis. *p < 0.05; **p < 0.01.

We then tested whether these findings persisted after accounting for additional factors that may have influenced NfL levels. First, we adjusted for the impact of CNS disease and degradation of central nervous tissue on NfL levels. To do so, we made use of paired CSF samples and calculated a ratio between sNfL levels and CSF‐NfL levels for each patient. Analysis using the ratio between sNfL and CSF‐NfL indicated a significant positive correlation between acute axonal damage scores and NfL_serum/CSF_ ratios (*r* = 0.5 [0.2–0.7]; *p* = 0.003; *n* = 30), but no correlation between parameters of chronic axonal loss and NfL_serum/CSF_ ratios (NF+ area: *r* = −0.03 [−0.4 to 0.3], *p* = 0.9, *n* = 29; density of myelinated fibers: *r* = −0.1 [−0.5 to 0.3], *p* = 0.6, *n* = 29) (Figure 4).

Second, we factored in patient age by calculating *Z*‐scores reflecting deviation from age‐specific reference values for each measurement [23]. Calculating age‐specific *Z*‐scores for sNfL levels indicated a significant correlation with acute axonal degeneration scores (*r* = 0.4 [0.1–0.7]; *p* = 0.01; *n* = 34). Parameters of chronic axonal loss showed discrepant results. Whereas there was no significant correlation between the density of myelinated fibers and age‐adjusted sNfL levels (*r* = −0.2 [−0.5 to 0.2] *p* = 0.3; *n* = 33), there was a significant negative correlation between the fascicular area covered by NF+ staining and age‐adjusted sNfL levels (*r* = −0.4 [−0.6 to −0.02]; *p* = 0.04; *n* = 32) (Figure 4).

In a secondary combined analysis restricted to patients without CNS disease, there was no significant correlation between acute axonal degeneration scores and age‐adjusted sNfL levels (*r* = 0.3 [−0.2 to 0.6]; *p* = 0.22; *n* = 25), no significant correlation between the density of myelinated fibers and age‐adjusted sNfL levels (*r* = −0.3 [−0.6 to 0.1]; *p* = 0.14; *n* = 24) and no significant correlation between NF+ area and age‐adjusted sNfL levels (*r* = −0.3 [−0.6 to 0.1]; *p* = 0.2, *n* = 23). In the restricted analysis, no significant correlation was detected between acute axonal degeneration scores and unadjusted sNfL levels (*r* = 0.4; [−0.04 to 0.7]; *p* = 0.07; *n* = 25), but a significant correlation between the density of myelinated fibers and unadjusted sNfL levels (*r* = − 0.4 [−0.7 to −0.02]; *p* = 0.03; *n* = 24).

## 4 DISCUSSION

In this comparison of the histopathological features of sural nerve injury and matched serum NfL levels in a cohort of patients that underwent sural nerve biopsy, we found a significant positive correlation between sNfL levels and acute axonal degeneration. In contrast, we did not find a correlation between sNfL levels and parameters of chronic axonal loss.

The studied cohort reflects typical clinical constellations in the context of sural nerve biopsy with a particular focus on patients with vasculitic neuropathy. Our findings are consistent with previous studies reporting elevated NfL levels in patients with active vasculitic neuropathy compared to vasculitis patients without neuropathy [7], as well as data showing that sNfL increases during active disease [7]. Taken together with our histopathological assessments, these findings suggest rapid release of NfL from axons within peripheral nerve tissue into the bloodstream after ischemic injury. A consequent decline of NfL levels during remission, as demonstrated by Bischof et al. [7], would explain the absence of a correlation between the extent of chronic axonal loss and NfL levels in our cohort. In line with previous electrophysiological studies on patients with chemotherapy‐induced neuropathy [18], we did find a moderate correlation between age‐adjusted NfL levels and the percentage of area covered by neurofilament. This discrepancy suggests that the usefulness of NfL as a predictor of disease severity needs to be further investigated at distinct timepoints in disease progression as well as in different disease entities. Nonetheless, considering the entirety of our analyses, our data do not support a meaningful association between chronic axonal loss and NfL levels.

Consequently, our findings mainly support the relevance of NfL release in active disease. This is illustrated by the strong association between NfL serum levels and the degree of acute axonal degeneration, most evident in patients with minimal (0%–20%) and severe (60%–80%) acute axonal damage scores (Figure 3). Four of five patients with severe acute axonal degeneration and correspondingly elevated NfL levels in serum and tissue in our cohort were diagnosed with subacute vasculitis (P2, P3, P7, and P9; Table 1). However, other studies have reported an acute release of NfL from peripheral nerve tissue in other disease entities such as Guillain‐Barré syndrome as well [17]. Taken together, although not specific to vasculitic neuropathy, measuring NfL in serum might be particularly useful for diagnosing acute disease and monitoring disease activity during follow‐up.

On a broader scope, disease specificity for biomarkers in vasculitis could be improved by using a combination of different biomarkers [29, 30]. From a histopathology perspective, biomarkers relating to vascular damage are of particular interest because they match our histopathological understanding of vessel destruction as the key diagnostic criterium of vasculitis. Again, this is illustrated by our patient cohort, in which perivasculitis is a common but non‐specific finding in patients with a clinical diagnosis of vasculitis (Table 1). In that respect, endothelial microparticles, circulated detached mature endothelial cells, and von Willebrand Factor (vWF) have been investigated in previous studies [31, 32]. In the future, combining NfL with biomarkers characterizing the immune response and vascular dysfunction in patients with vasculitic neuropathy could further improve diagnosis and disease monitoring.

Our study inherently comes with strengths and limitations. A strength of our study is the comprehensive histological workup to characterize nerve damage. To our knowledge, this study is the first study in this context to build on nerve biopsy tissue as the gold standard for nerve assessment. Also, our combination of established manual assessments with validated automated analyses (including a novel supervised‐learning‐based approach) promotes internal validity.

However, the study is limited by analysis of a relatively small clinical cohort because of limited availability of matched biosamples. It is also likely that factors such as renal function and CNS damage impact circulating sNfL levels [23, 33]. Among these factors, CNS damage is expected to have the most significant impact on NfL levels, as CSF NfL levels have been shown to correlate with blood NfL levels [34, 35]. In our cohort, nine patients presented with CNS‐disease (Table 1), and restricted analysis indicates that these patients contribute to the observed effect. Additionally, our adjustment for CNS damage based on serum/CSF NfL ratios relies on the assumption that CSF NfL values capped at the maximum detectable limit do not dramatically exceed this threshold. Nevertheless, high serum to CSF ratios are observed in patients both with and without CNS‐disease in our cohort and suggest that, in these cases, sNfL is released predominantly from the peripheral rather than the central nervous system [17]. By incorporating serum to CSF ratios, we were able to account for this key confounding factor without excluding patients with CNS conditions in our analysis. We believe this approach is superior to the secondary restricted analysis, and our sensitivity analysis supports the robustness of the results within a reasonable margin of uncertainty.

Another limitation of our study is that samples were collected at various stages of the underlying disease and with differing intervals between sample collection and nerve biopsy. However, NfL‐levels in serum and CSF are very stable both when cryoarchived [36, 37, 38, 39] and at room temperature [38, 39, 40], and the median time in our cohort between collection of serum/CSF and biopsy was only 4 days. Nonetheless, this variability remains a relevant limitation of our work. In particular, the timing and course of disease remain significant factors of uncertainty, especially regarding patients with an acute/subacute disease course where NfL‐levels can change with disease activity [7].

Lastly, when evaluating biomarkers in different biological compartments, variability in barrier function is an important consideration. It is well established in the central nervous system that blood–brain barrier integrity changes with disease [41]. Likewise, CSF diagnostics have long recognized the need to account for flow dynamics and the blood–CSF barrier in interpretation [42]. However, much less is known about the blood–nerve barrier and its impact on serum biomarker dynamics [43]. Finally, even direct flow of CSF to peripheral nerves needs to be considered as a factor that may influence our tissue‐level analysis [44].

Future prospective clinical studies are now needed to validate our findings, and these should control for concomitant CNS disease. Additionally, further research is required to clarify the role of blood–nerve‐barrier integrity in evaluating serum biomarkers for peripheral nerve degeneration. Finally, future research should incorporate complementary biomarkers such as peripherin, which is mostly expressed in the peripheral nervous system and therefore could be a more specific marker of peripheral nerve damage [45].

In conclusion, our findings support incorporating NfL as a biomarker of acute axonal degeneration in the diagnosis and monitoring of patients with vasculitic neuropathy. However, clinicians should take CNS disease into account when evaluating NfL levels and be aware that sNfL levels may also be elevated in other peripheral neuropathies. To improve specificity for vasculitic neuropathy, future studies should investigate markers that are more specific to peripheral nerve damage and combine NfL with markers reflecting pathophysiological hallmarks of vasculitis.

## AUTHOR CONTRIBUTIONS

SS designed the experiments, performed data acquisition and analysis, and drafted the manuscript. JM and WS supported histological analyses. NG established the automated NF70 image thresholding and segmentation approach. CS helped to establish the automated analysis of semithin sections. JK performed NfL measurements. CM provided expertise in the interpretation of NfL measurements. KR initiated the systematic biobanking of serum/CSF samples and provided help in collecting biosamples. JS facilitated access to resources used for the automated NF70 image analysis. FLH provided supervision and facilitated access to laboratory resources and technical support. PK and WS conceived of the initial idea of the study and supervised the project.

## FUNDING INFORMATION

The project was supported by a research grant from the John Grube Foundation e.V. and via the 22HLT07 NEuroBioStand project, which has received funding from the European Partnership on Metrology.

## CONFLICT OF INTEREST STATEMENT

Kemens Ruprecht received research support from Novartis, Merck Serono, German Ministry of Education and Research, European Union (821283‐2), Stiftung Charité, Guthy‐Jackson Charitable Foundation and Arthur Arnstein Foundation; received travel grants from Guthy‐Jackson Charitable Foundation; received speaker's honoraria from Virion Serion and Novartis; was a participant in the Berlin Institute of Health Clinical Fellow Program funded by Stiftung Charité. Péter Körtvélyessy served on advisory boards, received travel and accommodation reimbursement and speaker honoraria from Novartis, Alector, Roche Diagnostics, Biogen, Eli Lilly, EISAI, Biogen, and Schering Consultation, receives research funding from Roche Diagnostics.

## Supporting information


**Data S1.** Supporting Information.

## Data Availability

The data that support the findings of this study are available from the corresponding author upon reasonable request.
