# Supplementary material for: Neurofilament light chain as a marker of peripheral nerve damage in vasculitic neuropathy? A cross‐compartmental correlation analysis in patients undergoing nerve biopsy
Source: Brain Pathol. 2025 Sep 11;36(1):e70038. doi: 10.1111/bpa.70038 (PMC12695682; doi:10.1111/bpa.70038)
Supplement: Supplementary file 1 — Data S1. Supporting Information. [file BPA-36-e70038-s001.docx]

#
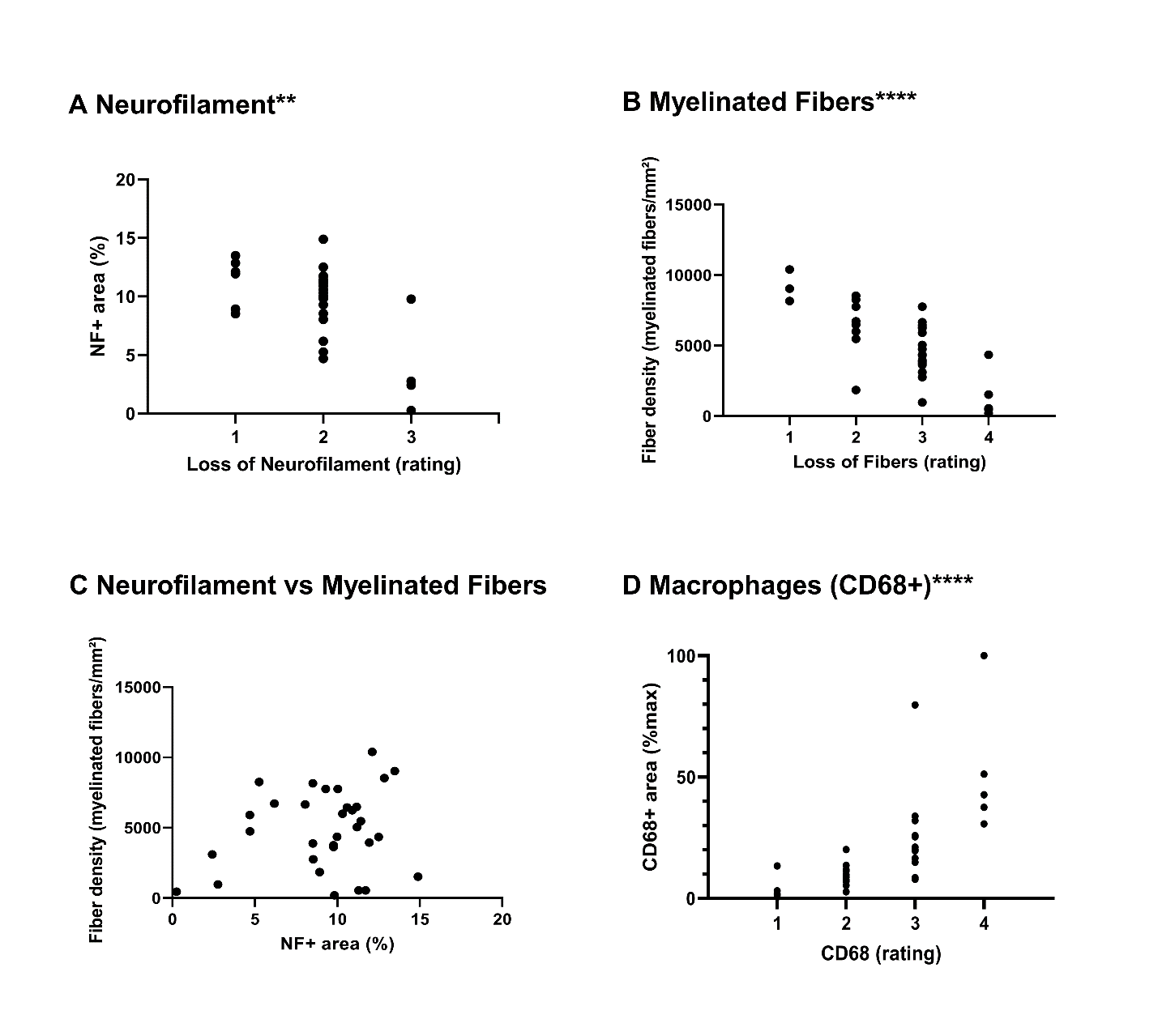
Supplementary Material 1

Validation experiments

(A/B/D) We performed semiquantitative assessments to validate our automated analysis. (A) We rated loss of neurofilaments on a scale from 0 (no loss of neurofilaments) to 5 (severe loss of neurofilaments). Ratings were then synthesized into 3 categories (1: no/mild loss of neurofilaments; 2: moderate loss of neurofilaments; 3: severe loss of neurofilaments). Semiquantitative assessment showed a significant correlation with the automated analysis (Spearman rank correlation, one-tailed; r= -0.5 [-0.7 to -0.2]; p = 0.002; n=33). (B) Automated detection of myelinated fibers correlates with a semiquantitative ranking of fiber loss (Spearman rank, one-tailed; r=-0.8 [-0.9 to -0.6]; p<0.0001; n=34 (C) Density of myelinated fibers and the percent area covered by NF70 as detected by automated analyses did not correlate with each other (Spearman rank, one-tailed; r=0.2 [-0.2 to 0.5]; p = 0.2; n=32). (D) Macrophage infiltration was ranked on a scale from 1 (no relevant macrophage infiltration) to 4 (extensive macrophage infiltration). Semiquantitative ranking showed a significant correlation to the automated analysis of CD68+ staining (Spearman rank correlation, one-tailed; r=0.8 [0.7 to 0.9]; p < 0.0001; n=33).


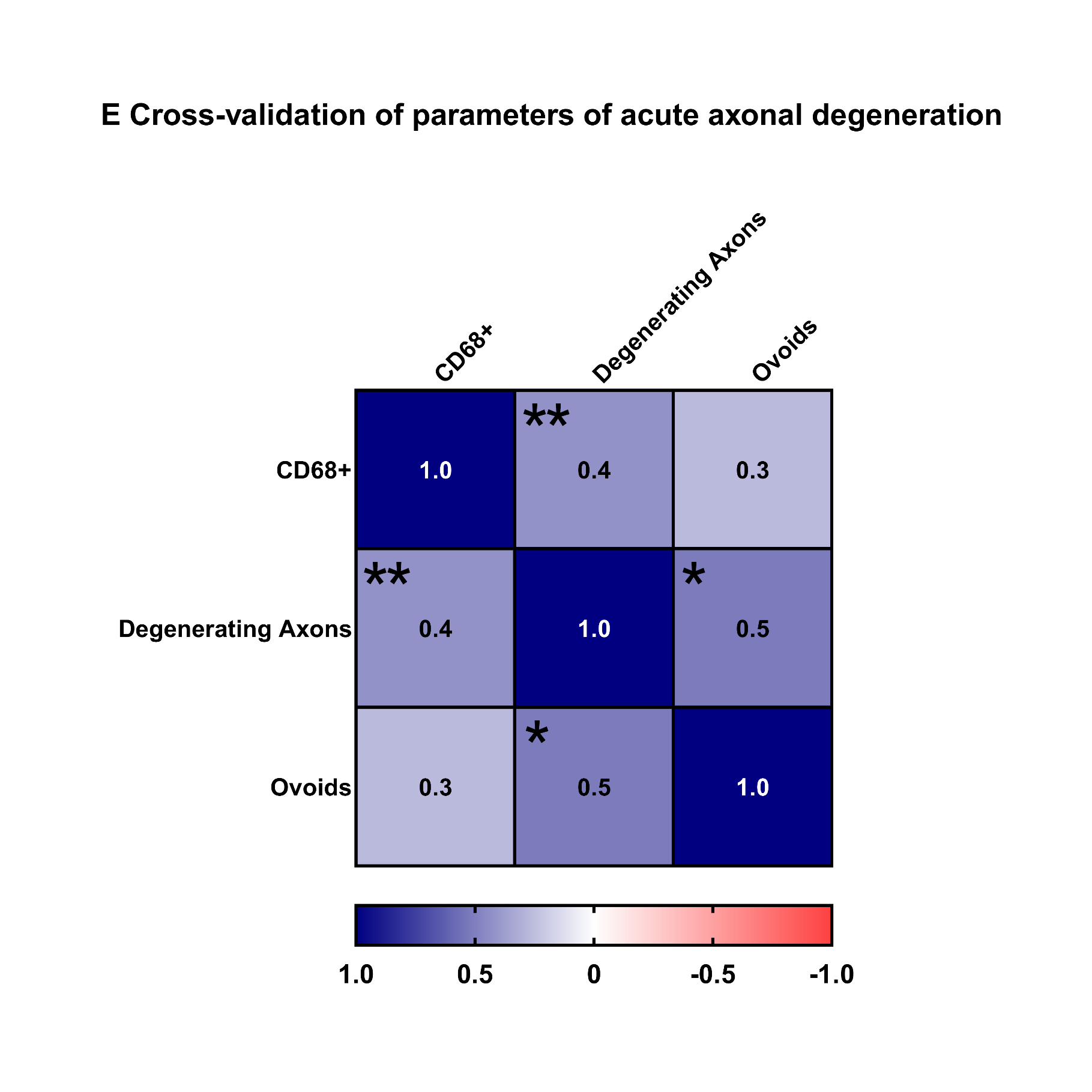


(E) Individual parameters of acute axonal damage correlate with each other (Spearman rank correlation, one-tailed). * p < 0.05; **p < 0.01. ****p < 0.0001.

#
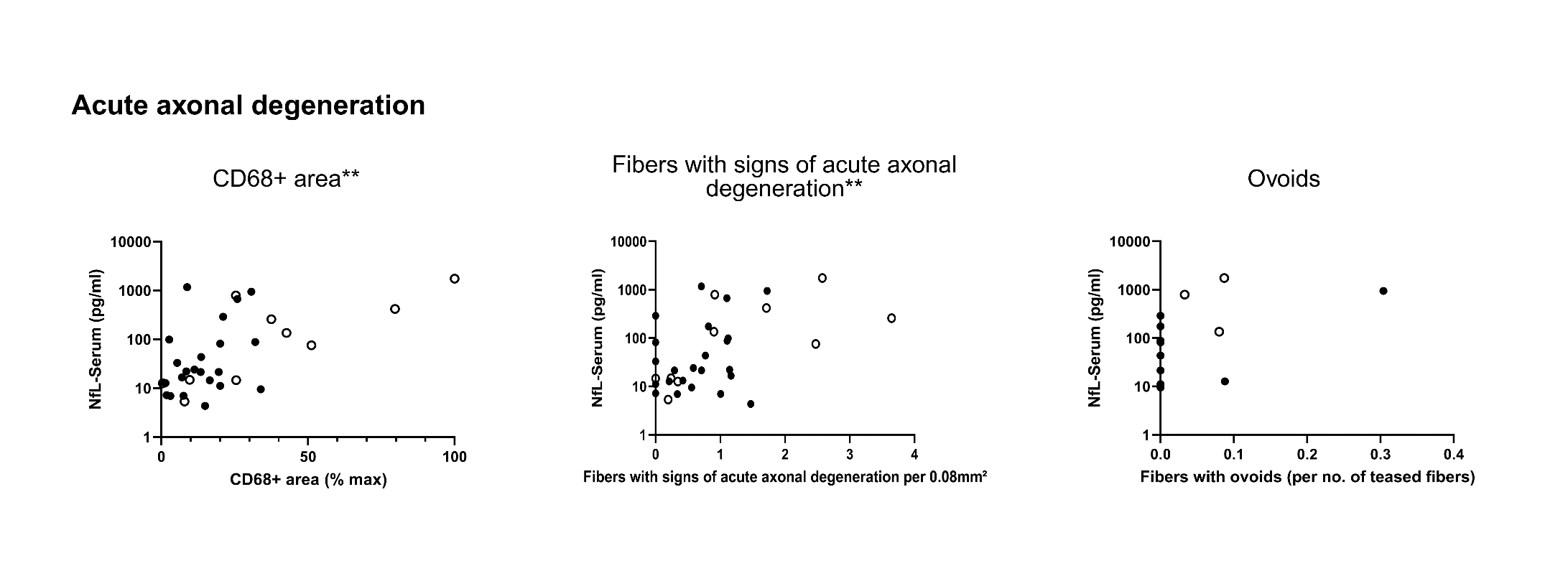
Supplementary Material 2

Separate analysis of parameters of acute axonal degeneration

For analysis, parameters of acute axonal degeneration were combined into a single composite score. The scatterplots display data points for each parameter individually. Unfilled dots represent data points from patients with a clinical diagnosis of vasculitis. The value 0.08mm² corresponds to the average fascicle area in our dataset. For analysis, we used Spearman rank correlation (two-tailed). * p < 0.05; **p < 0.01.

# Supplementary Material 3

## Units of measurement

|  | NF+ area | Fiber Density | CD68 | Degenerating Axons | Ovoids |
| --- | --- | --- | --- | --- | --- |
| Unit | % fascicular area covered by NF70+ staining | Density of myelinated fibers per mm² | % Maximum of CD68+ staining (per fascicular area; averaged across longitudinal and transverse data set) | No. of fibers with morphological signs of acute axonal degeneration (per average fascicle area) | Teased fibers with ovoids (per total no. of teased fibers) |

## NfL-Serum (unadjusted)

|  | NF+ area | Fiber Density | CD68 | Degenerating Axons | Ovoids |
| --- | --- | --- | --- | --- | --- |
| r | -0.2 (-0.5 to 0.1) | -0.3 (-0.6 to 0.1) | 0.5 (0.2 to 0.8) | 0.4 (0.1 to 0.7) | 0.5 (-0.1 to 0.8) |
| p | 0.23 | 0.08 | 0.001** | 0.009** | 0.11 |
| n | 32 | 33 | 32 | 33 | 13 |

## NfL-Serum (age-adjusted/Z-Scores)

|  | NF+ area | Fiber Density | CD68 | Degenerating Axons | Ovoids |
| --- | --- | --- | --- | --- | --- |
| r | -0.4 (-0.6 to -0.02) | -0.2 (-0.5 to 0.2) | 0.5 (0.2 to 0.7) | 0.3 (-0.01 to 0.62) | 0.5 (-0.1 to 0.8) |
| p | 0.04* | 0.31 | 0.004** | 0.05* | 0.07 |
| n | 32 | 33 | 32 | 33 | 13 |

## NfL Serum/CSF Ratio (adjustment for CNS disease)

|  | NF+ area | Fiber Density | CD68 | Degenerating Axons | Ovoids |
| --- | --- | --- | --- | --- | --- |
| r | -0.03 (-0.4 to 0.3) | -0.1 (-0.5 to 0.3) | 0.6 (0.2 to 0.8) | 0.5 (0.2 to 0.7) | 0.3 (-0.4 to 0.8) |
| p | 0.9 | 0.6 | 0.002** | 0.01** | 0.4 |
| n | 29 | 29 | 29 | 29 | 11 |

## NfL Tissue (% protein fraction)

|  | NF+ area | Fiber Density | CD68 | Degenerating Axons | Ovoids |
| --- | --- | --- | --- | --- | --- |
| r | -0.2 (-0.5 to 0.2) | -0.3 (-0.6 to 0.04) | 0.3 (-0.1 to 0.6) | 0.4 (0.1 to 0.7) | 0.2 (-0.4 to 0.7) |
| p | 0.3 | 0.07 | 0.09 | 0.01* | 0.6 |
| n | 31 | 31 | 31 | 31 | 13 |

# Supplementary Material 4


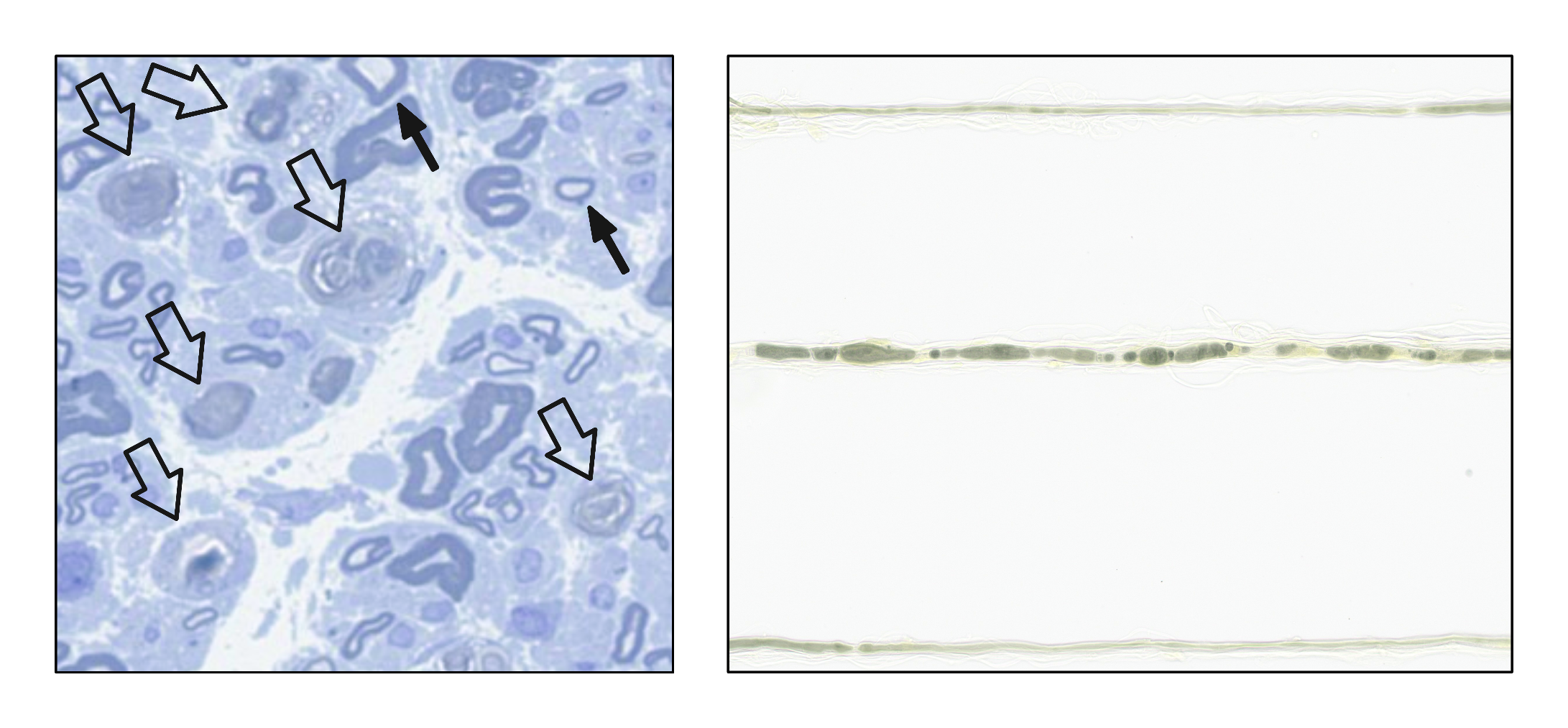


**(A)** The left image illustrates fibers classified as showing signs of acute axonal degeneration. Note the greyish discoloration of the Methylene-blue-stained tissue containing thin myelin remnants, occasional vacuolar alterations and absence of proper (centrally placed) axonal tissue as seen in surrounding fibers with a variably thick but integral myelin sheath (filled arrows). The right image shows teased fiber preparations, with the central fiber displaying ovoid formations reminiscent of Wallerian degeneration.


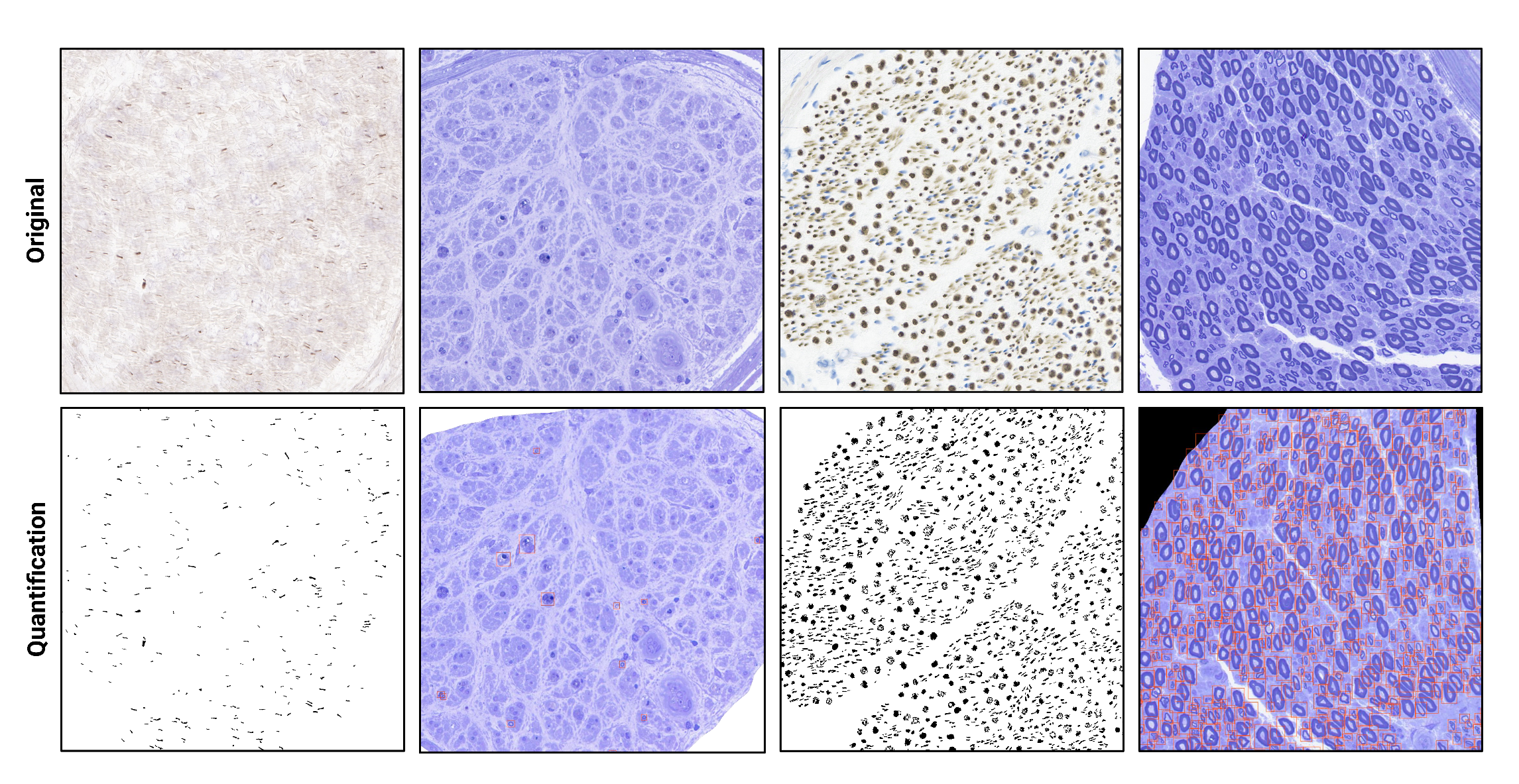
**(B)** The upper row shows the original images, and the lower row shows their quantification. Neurofilament staining was quantified based on intensity levels; black indicates the thresholded neurofilament-positive area. Myelinated fibers were quantified using a supervised learning–based approach, with orange rectangles marking automatically detected myelinated fibers.

# Supplementary Material 5

## Sensitivity Analysis (NfL Serum/CSF Ratio vs acute axonal damage score)

| Cap | 25,000 pg/ml | 33,825 pg/ml | 42,649 pg/ml | 50,000 pg/ml | 100,000 pg/ml |
| --- | --- | --- | --- | --- | --- |
| Rationale | Maximum validated detection limit | Midpoint between maximum validated limit and maximum extrapolated value | Maximum extrapolated value in our cohort | 2x the maximum validated detection limit | 4x the maximum validated detection limit |
| r | 0.5 [0.2 to 0.7] | 0.5 [0.1 to 0.7] | 0.4 [0.03 to 0.7] | 0.4 [0.02 to 0.7] | 0.3 [-0.1 to 0.6] |
| p | 0.003** | 0.01* | 0.03* | 0.03* | 0.1 |

Four CSF NfL measurements exceeded the maximum validated detection limit. For one of these, an extrapolated value of 42,649 pg/ml was provided, while the remaining three exceeded the extrapolation range and could not be quantified. To assess how different assumptions about these high values might influence the results, we performed a sensitivity analysis by repeating the correlation between NfL serum/CSF ratios and acute axonal damage scores using different upper cap values.
